# Microbial Antigens Stimulate Metalloprotease-7 Secretion in Human B-Lymphocytes Using mTOR-Dependent and Independent Pathways

**DOI:** 10.1038/s41598-017-04199-2

**Published:** 2017-06-20

**Authors:** Mohamed F. Ali, Harika Dasari, Virginia P. Van Keulen, Divi Cornec, George Vasmatzis, Tobias Peikert, Eva M. Carmona

**Affiliations:** 1 Thoracic Diseases Research Unit, Rochester, Minnesota 55905 United States; 2 Division of Pulmonary Critical Care and Internal Medicine, Rochester, Minnesota 55905 United States; 3 Department of Medicine and Biomarker Discovery, Center for Individualized Medicine Mayo Clinic and Foundation, Rochester, Minnesota 55905 United States

## Abstract

Metalloproteinases (MMPs) contribute to tissue remodeling and acute inflammation not only by degrading extracellular matrix proteins but also by controlling the influx of chemokines through the regulation and shedding of syndecans. B-lymphocytes, in addition to their well-known function as antibody producing cells, participate in the innate immune response by secreting inflammatory cytokines and chemokines. However, there is little information about the role of B-lymphocytes in the regulation of MMPs; consequently, herein we investigated whether activated human circulating B-lymphocytes contributed to the secretion of MMPs. We demonstrate that B-lymphocytes activated by un-methylated CpG motifs, found in bacterial DNA, and β-glucans, found in the cell wall of fungi, both induced MMP-7. Interestingly, while CpG-stimulated cells activated the mTOR pathway *via* TLR9 receptor to induced MMP-7, β-glucan-stimulated cells were mTOR-independent and used Dectin-1 receptor. B-lymphocytes did not seem to have a major role in the secretion of tissue inhibitors of metalloproteinases (TIMPs). However, secreted MMP-7 participated in the shedding of Syndecan-4 from the surface of B-lymphocytes. In conclusion, circulating human B-lymphocytes contribute to the regulation of the innate immune system by participating in the secretion of MMP-7 which in turn is important for the shedding of Syndecan-4 in response to infectious stimuli.

## Introduction

Matrix metalloproteinases (MMPs) are a group of enzymes that participate in the turnover and degradation of extracellular matrix proteins. High levels of MMPs are detectable during tissue inflammation and repair, suggesting their role in innate immune defense and wound healing^1^. Emerging evidence implicates MMPs in the regulation of non-matrix proteins such as cytokines, chemokines and antimicrobial proteins through regulation of syndecans^2^. Syndecans are type I transmembrane heparin sulfate proteoglycans that interact with a wide variety of ligands through the heparin sulfate chains located in the extracellular domains. MMPs participate in the regulation of syndecans by cleaving them off the cells surface. Upon shedding, the unbound syndecans will compete with the membrane-bound ones for the same ligands (cytokines, chemokines, collagens and extracellular matrix glycoproteins) thus participating in their regulation. Specifically, MMP-7 has been recently shown, in murine epithelial cells, to mediate the cleavage of syndecan-1 which regulates CXCL-1 (murine homolog of IL-8) influx and subsequent neutrophil recruitment to the injured epithelial surfaces^3^. MMP-7 also participates in the regulation of antimicrobial defensins^4^ and re-epithelization of airway and intestinal mucosal epithelial cells by controlling the shedding of E-cadherin^5^. To avoid exuberant MMP activation various tissue inhibitors of metalloproteinases (TIMPs) are in charge of their regulation by binding to their catalytic domains^6^. A balanced regulation between MMPs and TIMPs is therefore essential to control innate host defense and tissue repair/homeostasis.

Activated B-lymphocytes *via* pattern recognition receptor (PRR) are an important source of inflammatory cytokines and chemokines^7^. Furthermore, our group has recently demonstrated that interleukin 8 (IL-8) secreted by circulating B-lymphocytes activated by fungal cell wall carbohydrates (β-glucans) contribute to neutrophil recruitment to the site of inflammation^8^. However, while neutralization of IL-8 significantly decreased neutrophil chemotaxis, this reduction was not absolute; suggesting that other mechanisms or chemokines may also play a role. As MMPs are emerging as important controllers of chemokines and cytokines gradients by regulating syndecans proteins we sought to investigate the contribution of circulating B-lymphocytes to this important and not well understood inflammatory response.

Herein, we investigated the effects of PRR-mediated activation of B-lymphocytes on MMPs and TIMPs. Our work shows that B-lymphocytes activated by un-methylated bacterial DNA CpG motifs, and by fungal β-glucans both secrete primarily MMP-7 but neither stimulant had much effect on TIMPs. Interestingly, while CpG-mediated MMP-7 was TLR9 and mTOR-dependent, β-glucan-mediated MMP-7 was Dectin-1 dependent but mTOR-independent. Our findings demonstrate that the molecular pathways involved in MMP-7 regulation in B-lymphocytes differ among PRRs and their ligands. This differential regulation becomes important when considering therapeutic interventions that target MMP7. Furthermore, we identified MMP-7 as a sheddase for Syndecan-4 from the surface of B-lymphocytes.

## Material and Methods

### Reagents and antibodies

Endotoxin-free buffers and reagents were scrupulously used in all experiments. Curdlan, Zymosan, *Saccharomyces cerevisiae* and Laminarin were purchased from Sigma Chemical Co. (St Louis, MO). *Aspergillus fumigatus* β-glucan preparations were isolated as previously described^9^. To ensure that all glucan preparations were free of endotoxin prior to use in culture, Curdlan, Zymosan, *Aspergillus fumigatus* and *Saccharomyces cerevisiae* glucans were vigorously washed ten times with distilled physiological saline, incubated rotating overnight with polymyxin B (Sigma, St Louis, MO) at 4 °C, then vigorously washed again with distilled physiological saline. The final preparations were assayed for endotoxin with the limulus amebocyte lysate method using Pyrosate Rapid Endotoxin Detection Kit (Associates of Cape Cod, East Falmouth, MA) and found to consistently contain less than 0.25 EU/ml. Glucans were pulse sonicated 10 times using a Branson digital sonifier (VWR Scientific, Radnor, PA) at 35% amplitude immediately before addition to the cultures. The Erk inhibitor (PD98059), JNK inhibitor (SP600125), SYK inhibitor (Piceatannol), and NF-κB inhibitor (Bay11-7085) were all obtained from Calbiochem, Inc. (San Diego, CA). AP-1 inhibitor (SR 11302) was from R&D Systems, Inc. (Minneapolis, MN). MMP7 inhibitor (GM6001) was purchased from Cayman Chemical (Ann Arbor, Michigan) and Rapamycin was purchased from Selleck Chemicals (Houston, TX). Phosphorothioate-protected CpG oligonucleotide (5′-TCGTCGTTTTGTCGTTTTGTCGTT-3′) was commercially synthesized by Integrated DNA Technologies, Inc. Antibodies recognizing the cell signaling components ERK1/2, p-ERK1/2 and the mTOR pathway antibodies; mTOR, phospho-mTOR, S6K and phospho-S6K were from Cell Signaling Technology, Inc. (Danvers, MA). The neutralizing antibody for Dectin-1 was purchased from AbD Serotec (Raleigh, North Carolina) and Isotype control antibody was from R&D Systems, Inc (Minneapolis, MN). Recombinant human MMP7 was from EMD Millipore (Billerica, MA), MMP7 antibody was purchased from Abcam (Cambridge, MA) and Syndecan-4 antibody was purchased from Santa Cruz Inc. (Dallas, Texas). All other reagents were obtained from Sigma-Aldrich (St. Louis, MO) unless specified otherwise.

### Leucocyte Isolation and Culture

All methods were carried out in accordance with relevant guidelines and regulations. Human B-Lymphocytes were isolated as previously described^8^. Briefly, B-lymphocytes were isolated from acid citrate dextrose anticoagulated blood obtained from de-identify healthy volunteer platelet donors^10^ in accordance with the institutional review board (IRB) guidelines at the Mayo Clinic College of Medicine. Cells were isolated susing RossetteSep B-cell enrichment cocktail according to the manufacturer’s protocol (StemCell Technologies, Vancouver, Canada). The enriched B-lymphocyte population was repeatedly observed to contain an average of 93.1% ± 2.3% B-lymphocytes. To confirm that MMP-7 was not the result of other contaminating leukocytes in the enriched B-lymphocyte population, we performed an additional cell sorting of CD19-positive cells using a mouse anti-human CD19-APC (Miltenyi Biotec, San Diego, CA). The sorting was done using a FACSAria II SORP flow cytometer running FACSDiva v6.1.3 software (BD Biosciences, San Jose, CA). Cells were maintained in RPMI 1640 supplemented with 10% heat-inactivated fetal bovine serum and 1% Antibiotic-Antimycotic solution (Life Technologies, Grand Island, NY). Human peripheral neutrophil (PMNs) were isolated from heparinized whole blood (100 ml) that was collected from healthy human donors (This was approved by the institutional review board at the Mayo Clinic College of Medicine under the IRB-14005305. Informed consent was obtained from all subjects). The blood was layered over a Polymorphprep resolving media (Accurate Chemical, Westbury, NY) and spun at 500 × g for 15 min. The PMN rich fraction was collected and the RBCs lysed with Red Blood Cell Lysis Buffer. The PMNs were washed twice with phosphate buffered saline (PBS), and then resuspended in RPMI-1640 supplemented with 10% heat-treated fetal bovine serum and 1% Antibiotic-Antimycotic. For the experiments where neutrophils, B-lymphocytes and PBMC depleted of B-lymphocytes were used, the B-lymphocytes were isolated by CD19+ selection, as previously described. The flow through was labeled as PBMC depleted of B-lymphocytes. All the cell subtypes were from same donor.

### FACS for B-lymphocytes subtype analysis

Cells were counted, and 5 × 10^5^ cells/tube were incubated on ice for 30 minutes with cocktail of different antibodies (anti-CD20-APC-Alexa Fluor 700, Anti-Human IgD-APC, anti-CD27-PC7, anti-CD38-PC5.5, all from Beckman Coulter, Inc.) then washed, fixed in 1%-PFA PBS and analyzed by FACS (Canto X, BD Biosciences). For each experiment, unstained cells as well as single color controls were analyzed to check for individual compensations between fluorochromes. FACS data were analyzed and graphed using KALUZA and FlowJo softwares. Among CD20^+^ B-cell subsets were defined as follows: naïve (IgD^+^CD27^−^), unswitched memory (IgD^+^CD27^+^), switched memory (IgD^−^CD27^+^) and plasmablasts (CD38^++^CD27^++^).

### MMP-7 ELISA

B-lymphocytes (4 × 10^5^ cells/well in 96-well plates) were cultured with 1 μg/ml of CpG or with 200 μg/ml of Curdlan (Curd), 200 μg/ml Zymosan (Zym), 200 μg/ml *Saccharomyces cerevisiae* β-glucan (SacG) or 200 μg/ml *Aspergillus fumigatus* β-glucan (AspG) in culture medium for 24 hours unless otherwise indicated. Cell supernatants from 24 hours stimulated cells were then analyzed for MMP-7 production using human MMP-7 DuoSet Elisa kit from R&D Systems, Inc. (Minneapolis, MN) according to the manufacturer’s instructions. Inhibitors (30 μM PD98059, 20 μM SP600125, 10 μM Bay11-7085, 10 μM SR11302 or 1 mg/ml Laminarin) and neutralizing antibodies (5 μg/ml anti-Dectin-1 IgG or mouse IgG) were incubated with B-lymphocytes for 1 hour prior to addition of CpG or β-glucan preparations. All inhibitors were dissolved in dimethyl sulfoxide (DMSO) unless otherwise indicated. Solvents were added to all treatments to assure that solvents concentrations are the same in all treatment conditions.

### RNA isolation and real-time qPCR analysis

Total cellular RNA was isolated from B-lymphocytes using RNeasy Plus Universal Mini Kit (Qiagen) according to the manufacturer’s instructions. For cDNA synthesis, total RNA concentrations and purity were determined using a Nanodrop ND-1000 spectrophotometer, and 1 µg RNA was used in a 20 µl reaction mixture using a Verso cDNA Synthesis Kit (Thermo scientific). Quantitative real-time PCR was performed in 10 µl reaction volumes in a 96-well plate using 2 µl of diluted cDNA with SYBR Premix Ex Taq (Clontech Laboratories, Inc.) on a ViiA7 Real-Time PCR Detection System (Life technologies). The data were analyzed with ViiA7 software, version 1.2.4 (Life technologies). Relative transcript expression of each target gene was determined using the comparative Ct method, and the amounts of various cytokines and receptor transcripts were normalized to GAPDH transcripts in the same cDNA samples. The results were expressed as % of the target gene relative to that of GAPDH and plotted as the mean ± standard error of the mean (SEM). The primer pairs used in these assays are listed in Supplement 1.

### Microarray analysis

Microarray analysis was conducted according to manufacturer’s instructions for the Affymetrix 3′ IVT Plus kit (Santa Clara, CA). Briefly, RNA quality was assessed by Agilent Bioanalyzer (Santa Clara, CA). Reverse transcription to second strand cDNA was generated from 100 ng of high quality total RNA. Subsequently, the products were *in vitro* transcribed to generate biotin-labeled cRNA. The IVT products were then bead-purified (Affymetrix), fragmented, and hybridized onto Affymetrix Prime GeneChips® at 45 °C for 16 hours. Subsequent to hybridization, the arrays were washed and stained with streptavidin-phycoerythrin, then scanned in an Affymetrix GeneChip® Scanner 3000 (Santa Clara, CA). All Affy array experiments were carried out at the Mayo Clinic Genome Analysis Core. Control parameters were confirmed to be within normal ranges before normalization and data reduction was initiated.

### Cellular viability

Cell viability was assessed using the XTT Cell Proliferation Kit II (Roche Molecular Biochemicals, Indianapolis, IN) according to the manufacturer’s protocol. This assay measures the conversion of sodium-3′-[1-(phenylaminocarbonyl)-3,4-tetrazolium]-bis(4-methoxy-6-nitro) benzenesulfonic acid hydrate (XTT) to a formazan dye through electron coupling in metabolically active mitochondria using the coupling reagent N-methyldibenzopyrazine methyl sulfate. Only metabolically active cells are capable of mediating this reaction, which is detected by absorbance of the dye at 450–500 nm. Briefly, 50 μl of the XTT labeling mixture was added to the 100 μl of growth medium containing B-lymphocytes and different concentrations of the inhibitors. The XTT labeling mixture was added in parallel samples 24 hours after the addition of the various inhibitors. A set of blanks were also included that did not contain cells and were treated identically as the normal samples. In addition, a set of solvent controls was also included for each inhibitor. Absorbance was measured at 6 hours after XTT addition. All treatments were performed in 3 replicates. Only inhibitor concentrations that elicited less than 20% net toxicity were used in these assays.

### Preparation of Cell Lysates, Electrophoresis and Immunoblotting

Total cellular proteins were obtained from B-lymphocytes following the described culture conditions. Briefly, the cells were washed with cold PBS twice and lysed in RIPA buffer (50 mM Tris-HCl pH 7.4, 15 mM NaCl, 0.25% deoxycholic acid, 1% NP-40, 1 mM EDTA) freshly supplemented with 1 mM phenylmethylsulfonyl fluoride (PMSF), mammalian protease inhibitor mixture, 10 mM Na Fluoride and 1 mM Na Orthovanadate. Cells were kept on ice for 15 minutes and then the lysates were centrifuged at 12,000 × *g* for 10 min at 4 °C. The resultant soluble supernatant contained total cellular protein. Protein concentrations were determined with the Bio-Rad protein assay (Hercules, CA) using BSA as the standard. Equal amounts of total cellular proteins were separated on SDS-10% polyacrylamide gels with Precision Plus Protein Dual Color Standards (Bio-Rad; Hercules, CA) being used as the molecular weight standards. Proteins were then transferred to Immobilon-P membranes (Millipore, Bedford, MA). When supernatants are analyzed, the high molecular weight part of the membranes were cut and stained with Ponceau S solution (Sigma, St Louis, MO). Membranes were then blocked at room temperature for 30 min with 1% BSA/TBST (TBS, pH 7.4, 1% BSA, 0.1% Tween 20) and incubated overnight at 4 °C in a blocking solution containing primary antibodies at the appropriate dilutions. After washing with TBST, the membranes were incubated with horseradish peroxidase (HRP)-conjugated secondary antibodies for 1 hour. Immunoreactive bands were detected with SuperSignal West PicoChemiluminescent Substrate from Thermo Scientific (Rockford, IL). Actin was used as loading control for the cell lysates and a non-specific protein for the cell supernatant). Band intensities were quantified using ImageJ software 1.48 v (National Institutes of Health, Bethesda, MD, USA), and the relative intensities was calculated by normalizing the intensity of the target protein to that of the loading control.

### Statistical analyses

All data are presented as the mean ± SEM, from at least three experiments from different biological donors. The data were first analyzed using one-way ANOVA and posttest Sidak’s comparisons unless otherwise indicated. Statistical analysis was performed using GraphPad Prism Version 6 (GraphPad Software, La Jolla, CA).

## Results

### MMPs and TIMPs expression in CpG-stimulated B-lymphocytes

**Figure 1:**
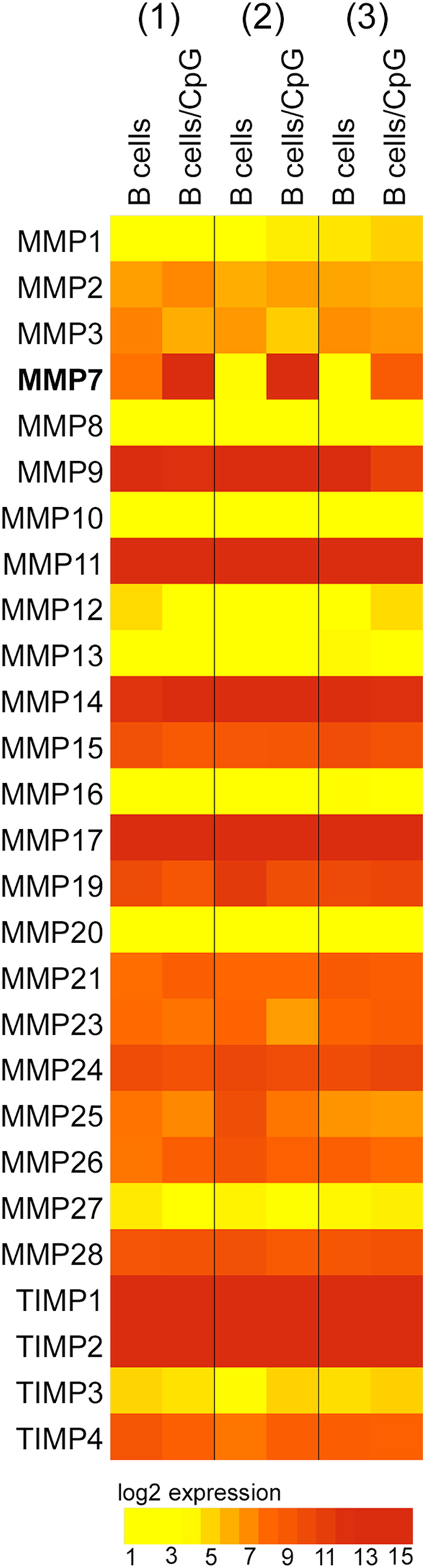
Heat map of MMPs and TIMPs expressed in human peripheral B-lymphocytes at baseline and after CpG stimulation. B-lymphocytes were isolated from peripheral blood of three different heathy donors. Cells were left untreated or stimulated with CpG for 72 hours. Gene expression profiles were generated using Affymetrix PrimeView chip from isolated RNA and analyzed using transcriptome console 3.0. Genes were considered increased from baseline if a change of at least 2-fold or greater was observed.

**Figure 2:**
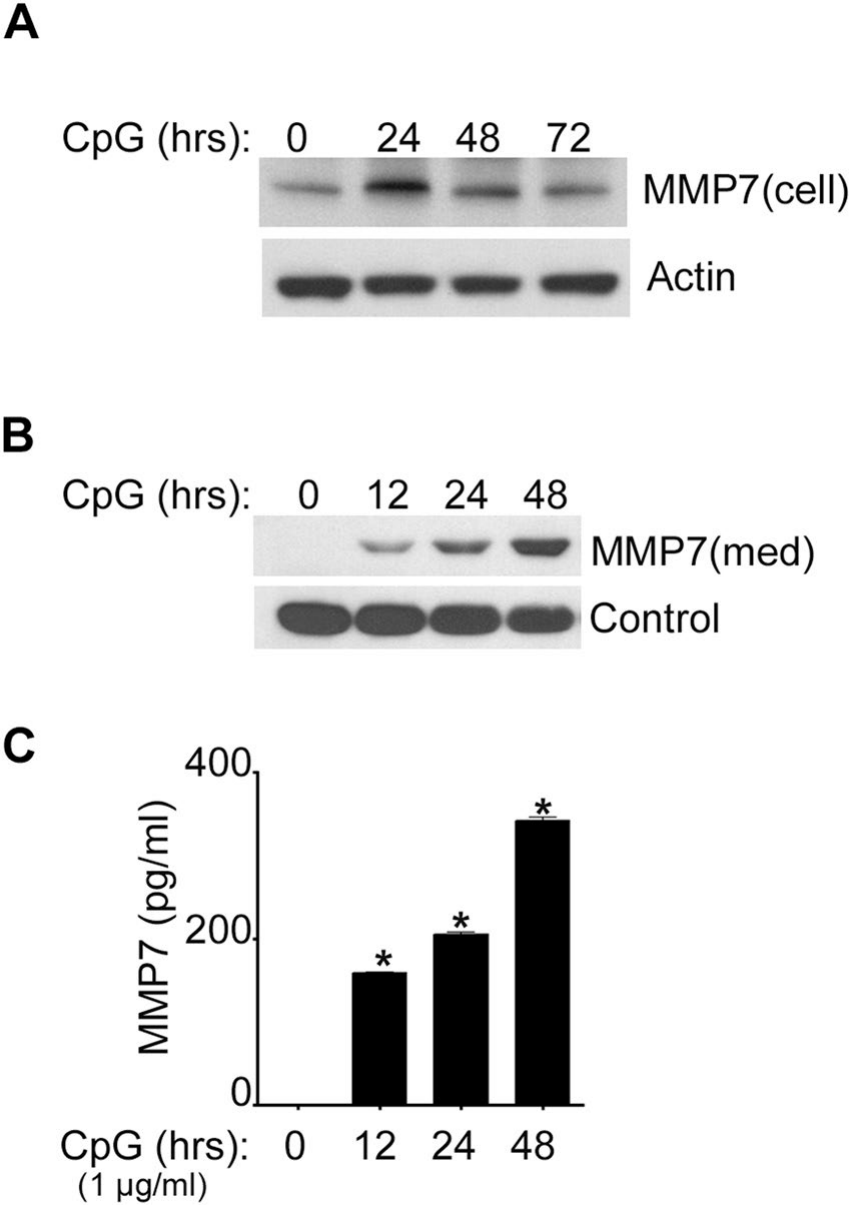
MMP-7 secretion by CpG-stimulated B-lymphocytes. MMP-7 was detected by immunoblotting in the cell lysate (cell) (**A**); and the cell supernatant (med) (**B**) of B-lymphocytes that were stimulated with 1 μg/ml of CpG as indicated. (**C**) MMP-7 concentration was measured by ELISA in the cell supernatant of CpG stimulated cells for different periods of time. Data are representative of at least three independent experiments. *p < 0.001.

To examine if human B-lymphocytes contribute to the circulating pool of MMPs and TIMPs, gene expression was analyzed on unstimulated and CpG-stimulated human peripheral B-lymphocytes using microarray technology. Except for MMP-7, treated and untreated B-lymphocytes had similar gene expression for the remaining MMPs and TIMPs (Fig. 1). MMP-7 was however, found to be upregulated in CpG-treated cells from different normal donors. Microarray data was further confirmed by qPCR (Supplement 2). Next, MMP-7 protein expression was measured in the cell lysates and the supernatants from circulating B-lymphocytes that were either isolated by negative selection or by flow cytometry cell sorting on CD19 positive cells. Levels of MMP-7 were found to be significantly elevated and accumulated in the cell supernatant in a time-dependent manner (Fig. 2 and Supplement 3). The levels of MMP-7 were found to be similar regardless of the isolation process confirming that B-lymphocytes are an important source of MMP-7. For the rest of the experiments we therefore elected to use negative selection as it is both time and cost-effective.

### The mTOR pathway regulates the secretion of MMP-7 in CpG-activated B-lymphocytes

**Figure 3:**
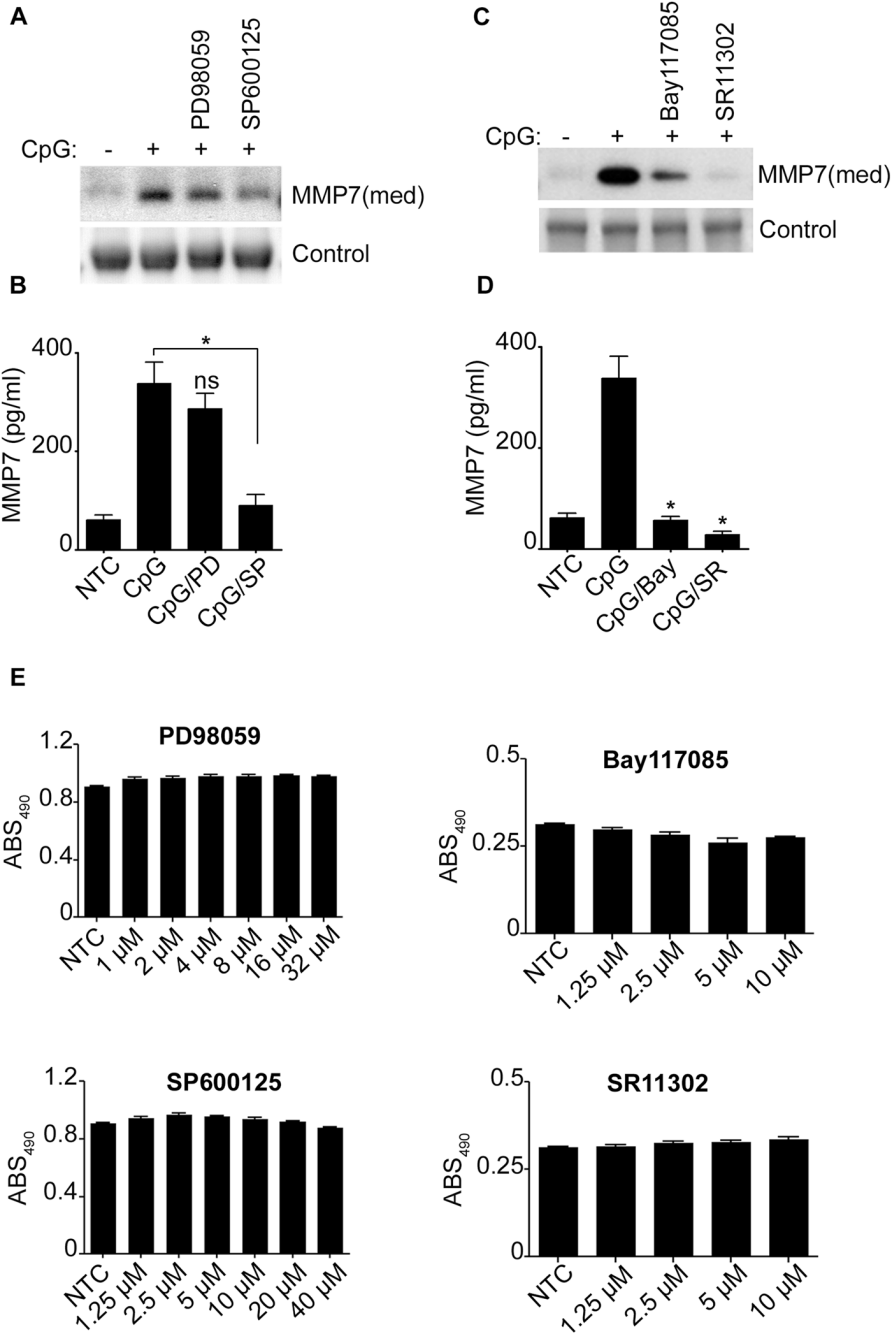
JNK and the transcriptions factors AP-1 and NF-κB participate in the regulation of MMP7 of CpG-Activated B-lymphocytes. MMP-7 was detected by immunoblotting (**A**,**C**) or by ELISA (**B**,**D**) in the cell supernatant (med) of unstimulated or CpG-stimulated cells. Cells were pretreated for one hour with the MAPK inhibitors, 30 μM PD98059 and 20 μM SP600125; or the transcription factor inhibitors, 10 μM Bay 117085 and 10 μM SR11302. (**D**) Cells were treated with different concentrations of the inhibitors as indicated. XTT was measured after 24 hours. Data are representative of at least three independent experiments. *p < 0.0001, ns, not significant (*p* > 0.05).

To further determine the molecular pathways that regulate MMP-7 and since CpG is a TLR9 agonist known to activate human B-lymphocytes *via* the Mitogen-activated protein kinases (MAPK) and c-Jun N-terminal kinase (JNK) but not extracellular signal-regulated kinases (ERK1/2) pathway^11^, we consequently explored the role of MAPK in the secretion of MMP-7. As anticipated, MMP-7 levels were significantly reduced in the presence of the JNK inhibitor (SP600125) but not the ERK inhibitor (PD98059) suggesting that only JNK participates in the regulation of MMP-7 (Fig. 3A and B). In addition, MMP-7 was significantly inhibited by a nuclear factor-κB (NF-κB) (Bay-117085) and an activator protein-1 (AP-1) (SR11302) inhibitor (Fig. 3C and D) confirming the contribution of the transcriptions factors NF-κB and AP-1 in CpG-mediated MMP-7 regulation.

**Figure 4:**
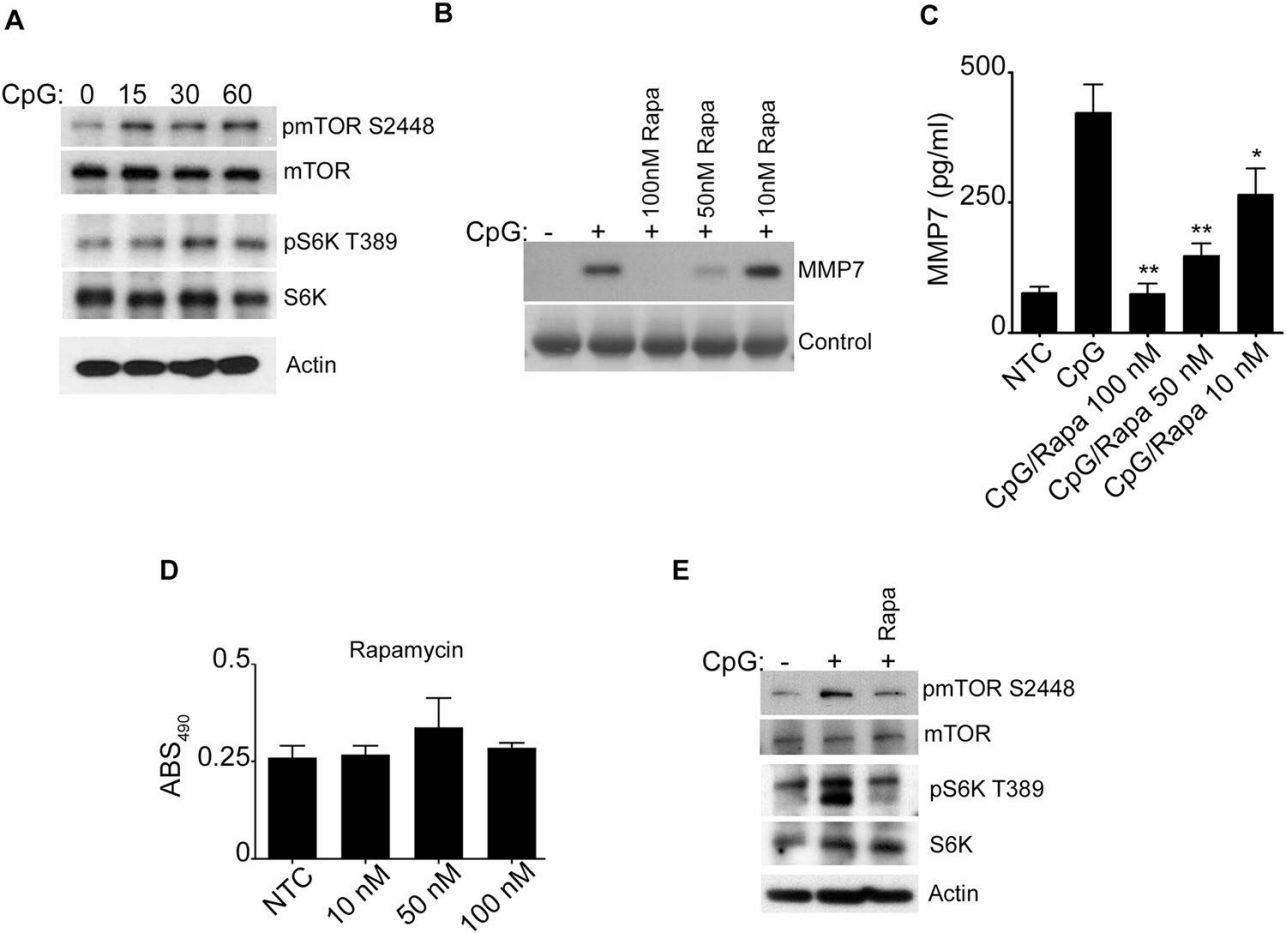
CpG-mediated MMP-7 activation by B-lymphocytes requires the mTOR pathway. (**A**) Phosphorylation of mTOR and pS6K was detected by immunoblotting in the cell lysate of CpG-stimulated cells. Rapamycin at the concentrations shown was used prior to stimulation, and then MMP-7 was measured by immunoblotting (**B**) and ELISA (**C**) in the cell supernatant. (**D**) XTT was measured after 24 hours in the presence of different concentrations of Rapamycin. (**E**) Phosphorylation of mTOR and pS6K was detected by immunoblotting in the cell lysate. Some cells were pretreated with (100 nM) Rapamycin prior to CpG stimulation. Total mTOR, S6K and actin were used as controls. Data are representative of at least three independent experiments. *p < 0.05, **p < 0.0001.

Because MMP-7 regulation by CpG activated B-lymphocytes appears to follow the classic TLR9 activation pathway and since TLR9 mediated type I interferon (IFN-α/β) secretion is regulated by the mammalian target of Rapamycin (mTOR) pathway in dendritic cells^12^, we investigated the role of mTOR in our B-lymphocyte system. First, we confirmed that CpG induced phosphorylation of mTOR and the mTOR-related protein S6K (Fig. 4A). Next, cultured cells were treated with increasing concentrations of Rapamycin, a well-known mTOR inhibitor blocking the MTORC1, prior to CpG stimulation. Cells showed significantly reduced MMP-7 secretion in the presence of the inhibitor (Fig. 4B and C) as well as decreased phosphorylation of mTOR and S6K (Fig. 4E) suggesting that mTOR-pathway is involved in the regulation of MMP-7 production by CpG stimulated B-lymphocytes.

### MMP-7 activation by β-glucans differs from CPG and is mTOR-independent

**Figure 5:**
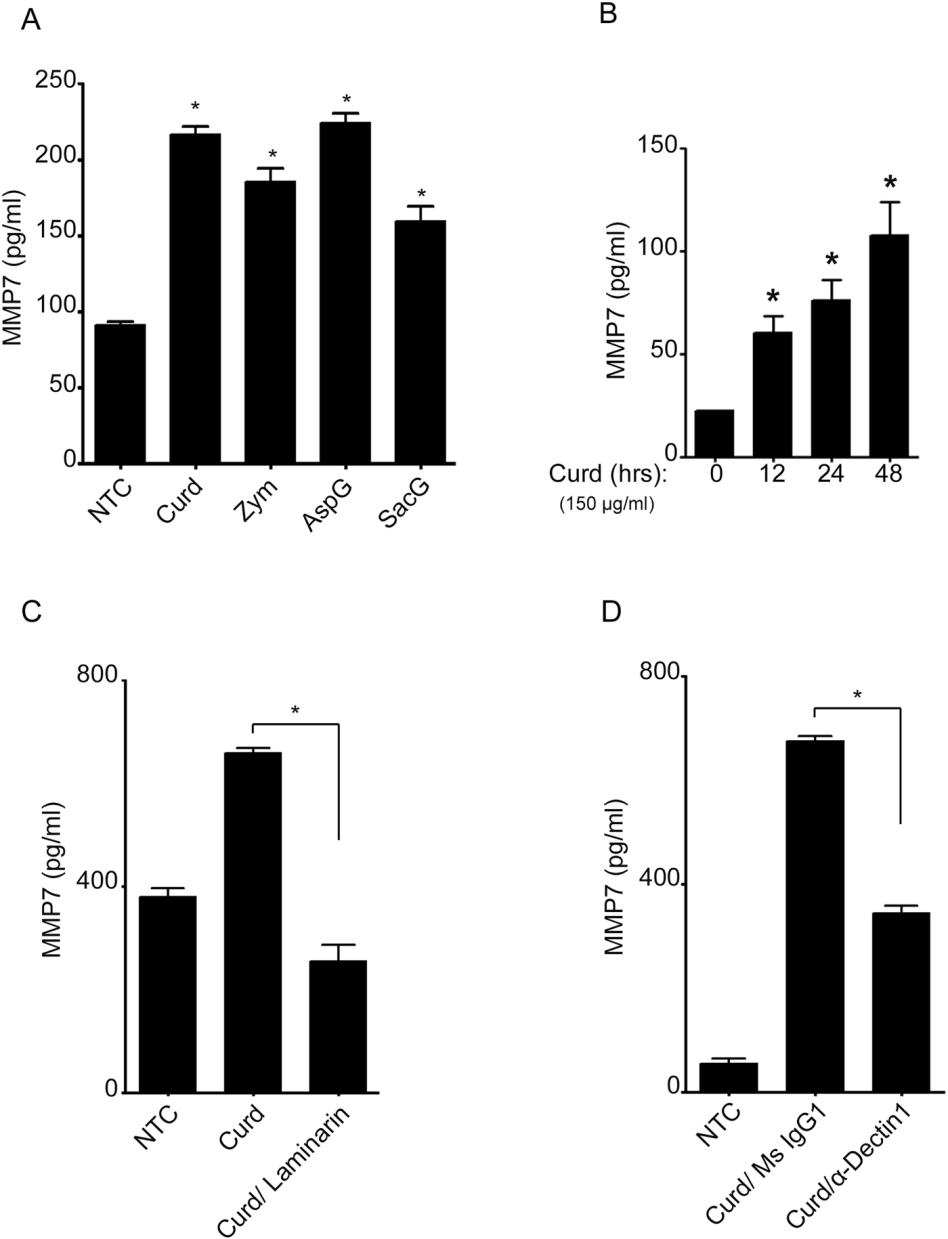
β-glucans induce MMP7 *via* Dectin-1. MMP-7 was measured by ELISA in the cell supernatant of B-lymphocytes after stimulation with different β-glucan preparations: Curdlan (Curd), Zymosan (Zym), *Aspergillus fumigatus* (AspG) and *Saccharomyces cerevisiae* (SacG) (**A**) and after stimulation with Curdlan for different periods of time (**B**). MMP-7 measured by ELISA of B-lymphocytes pre-incubated with laminarin (1 mg/ml) (**C**)**;** or anti-Dectin-1 antibody (**5** μg/ml) (**D**). Data are representative of at least three independent experiments. *p < 0.001.

**Figure 6:**
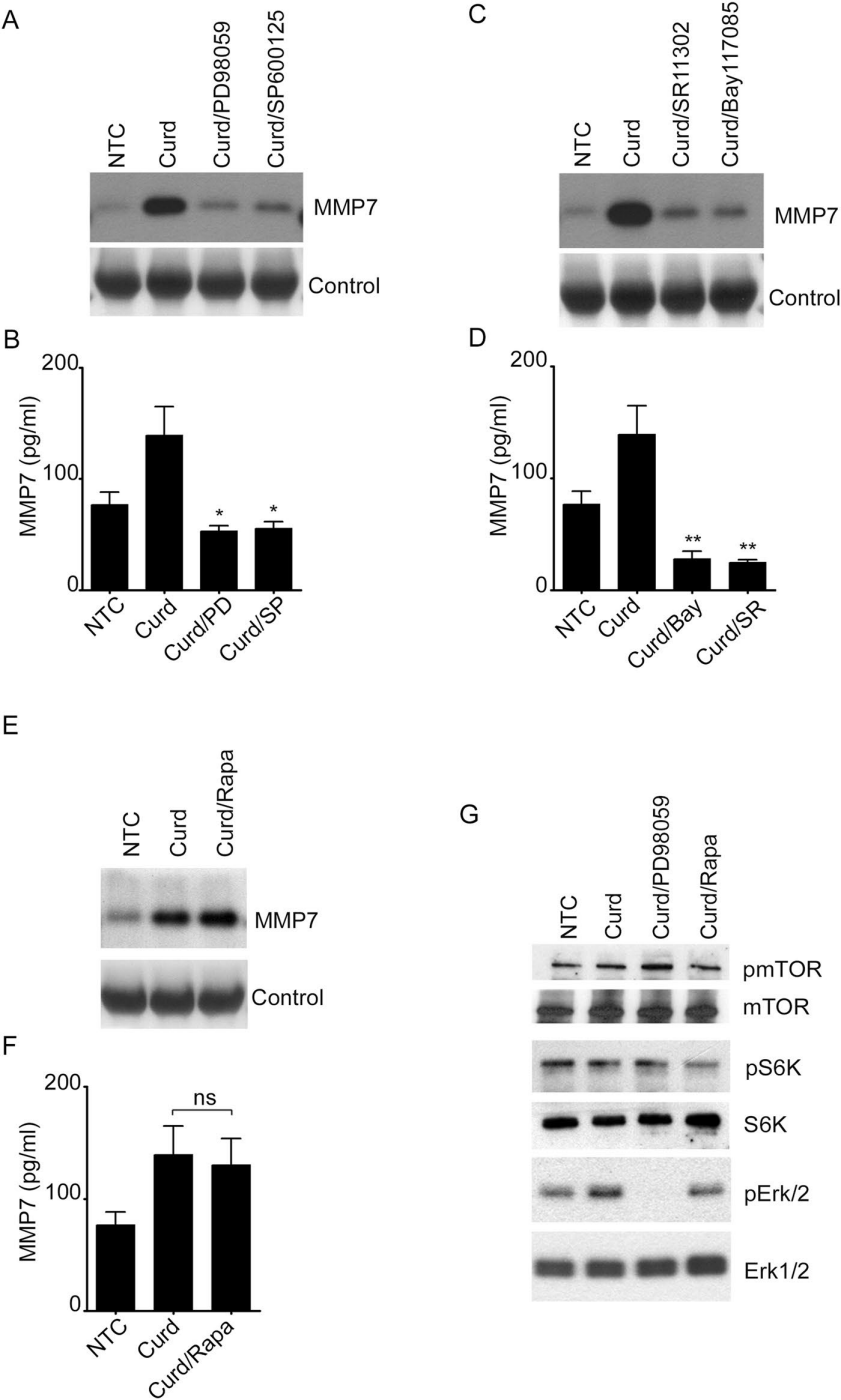
Curdlan induces MMP7 in an mTOR-independent manner. MMP-7 secretion was measured by immunoblotting (**A**,**C**,**E**) or ELISA (**B**,**D**,**F**) in the cell supernatant of unstimulated or Curdlan-stimulated cells. Cells were pre-incubated with 30 μM PD98059, 20 μM SP600125, 10 μM SR11302, 10 μM Bay 11-7085 and (100 nM) Rapamycin prior to Curdlan stimulation as indicated. (**G**) Phosphorylation of mTOR, S6K and ERK were detected by immunoblotting in the cell lysate of Curdlan-stimulated cells in the presence of 30 μM PD98059 and (100 nM) Rapamycin. Total mTOR, S6K and ERK were used as controls. Data are representative of at least three independent experiments. *p < 0.05, **p < 0.01, ns, not significant (*p* > 0.05).

To determine if the release of MMP-7 was a CpG/TLR9 specific response and since our previous work showed that B-lymphocytes in response to CPG and β-glucans produce two different cytokine profiles^8^, we examined the MMP-7 response to different particulate β-glucan preparations. Interestingly, MMP-7 was induced in comparable levels by all the β-glucans tested (Fig. 5A) and followed similar time course as CpG (Fig. 5B). To further explore the molecular mechanisms that regulate MMP-7 secretion in β-glucan stimulated cells and since our prior data using Curdlan, a well-established particulate 1-3 β-glucan, showed that Dectin-1 is one of the main receptors in Curdlan stimulated B-lymphocytes^8^, we investigated its participation in MMP-7. B-lymphocytes were pre-incubated with either laminarin (a soluble β-glucan form known to bind to Dectin-1 and act as a competitive inhibitor) or a specific Dectin-1 blocking antibody prior to stimulation with Curdlan. As shown in Fig. 5, MMP-7 levels were significantly reduced in the presence of both laminarin (Fig. 5C) and the Dectin-1 antibody (Fig. 5D), confirming its role in Curdlan-mediated MMP-7 secretion. Curdlan stimulation was also dependent on JNK and the transcription factors NF-κB and AP-1 (Fig. 6A–D). But, in contrast to CpG stimulation, MMP-7 was significantly reduced in the presence of the ERK inhibitor (Fig. 6A and B) and surprisingly, it was not affected by Rapamycin (Fig. 6E and F). Furthermore, Curdlan-stimulation failed to elicit an increase in the phosphorylation of mTOR and S6K while still increasing ERK1/2 phosphorylation, confirming that Curdlan mediated MMP-7 is independent of the activation of the mTOR pathway (Fig. 6G).

**Figure 7:**
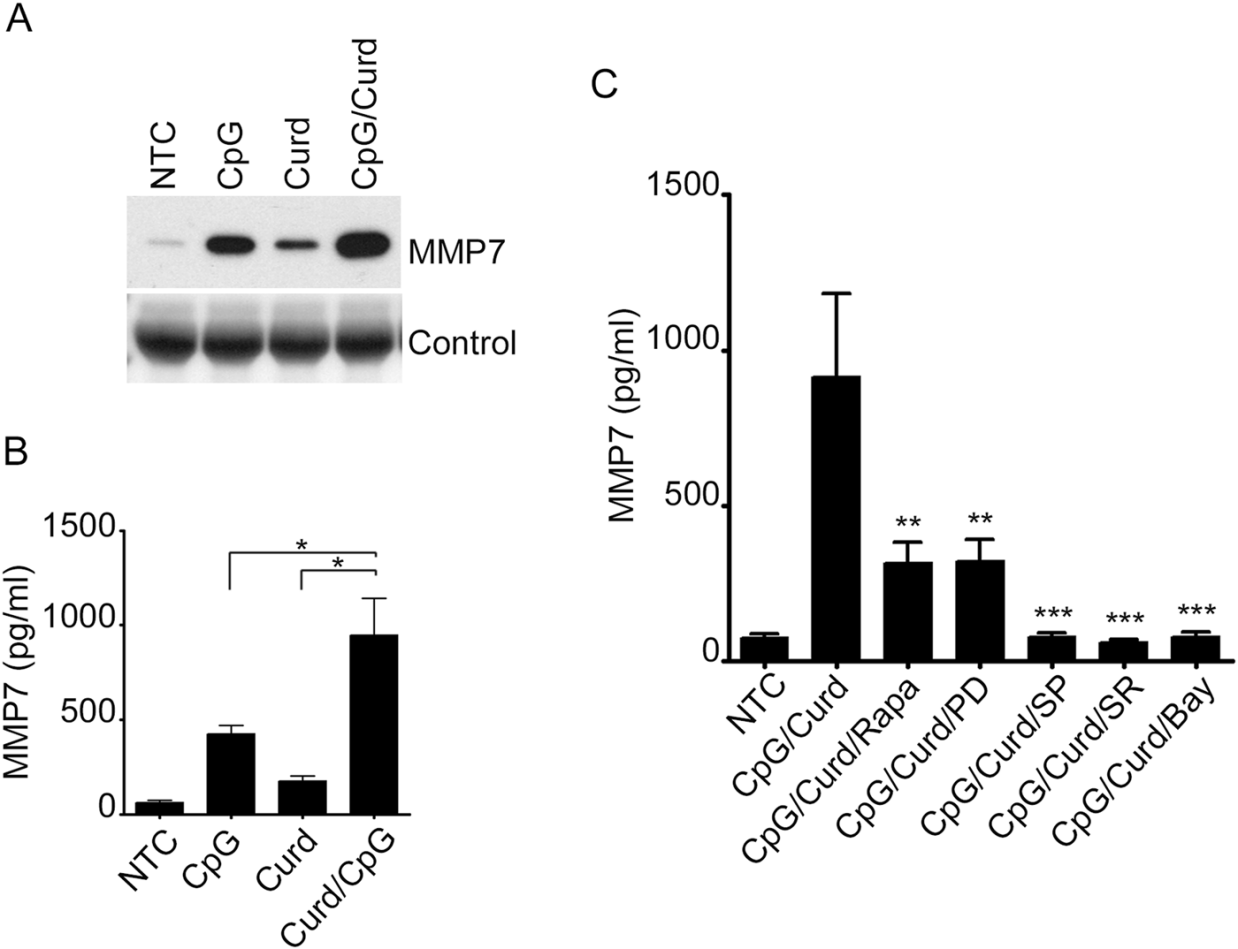
CpG and Curdlan have synergistic effect on MMP-7. MMP-7 was detected by Immunoblotting (**A**) or ELISA (**B**,**C**) in the supernatant of B-lymphocytes after stimulation with CpG, Curdlan and the combination of CpG and Curdlan overnight. Cells were pre-incubated with 30 μM PD98059 and the 20 μM SP600125, 10 μM SR11302, 10 μM Bay 117085 and (100 nM) Rapamycin prior to Curdlan stimulation as indicated. Data are representative of at least three independent experiments. *p < 0.05, **p < 0.01, ***p < 0.0001.

Interestingly, compared to CpG, Curdlan stimulation resulted in less MMP-7 expression and secretion, but combined stimulation resulted in a synergistic effect (Fig. 7A and B). As expected, this effect was significantly blocked by the inhibition of the JNK, NF-κB and AP-1 pathways (Fig. 7C). However, inhibition of ERK1/2 and the use of Rapamycin only resulted in partial inhibition of MMP-7 (Fig. 7C) supporting the presence of an mTOR-independent and -dependent stimulation triggered by Curdlan and CpG respectively.

Since MMP-7 was differently regulated by CpG and β-glucans, we next investigated if it was secreted by the same subpopulation of B-lymphocytes. As naïve and memory B-lymphocytes are the main subpopulations of B-lymphocytes in human peripheral blood, cells were sorted into CD20^+^CD27^−^ (naïve) and CD20^+^CD27^+^ (memory) cells prior to CpG and curdlan stimulation. MMP-7 was then assessed in the cell supernatant of each subtype. MMP-7 was significantly higher in naïve than memory cells in both CpG and Cudlan-stimulated cells (Supplement 4A,B). Other innate cells such as neutrophils and PBMCs depleted of B-lymphocytes did not show increase MMP-7 secretion in response to CpG or Curdlan (Supplement 4C).

### Role of the B cell receptor (BCR) and other TLRs in MMP-7 secretion

As B-lymphocytes express other TLRs as well as the B cell receptor (BCR), we next investigated if these receptors also contributed to MMP-7 secretion. First, we looked at the role of TLR2, important for the recognition of a wide range of microbial antigens, TLR3 as the main receptor for viral dsRNA and TLR5, as the main flagellin receptor and important for recognition of motile bacteria. Interestingly, with the exception of TLR2 which stimulated MMP-7 secretion, none of the other TLR tested resulted in a significant increase of MMP-7 (Supplement 5A). Next, we investigated the effect of BCR stimulation on MMPs and TIMPs and showed that BCR stimulation alone did not result in a significant increase in expression of any of the MMPs or TIMPs tested including MMP-7 (Supplement 5B,D). Remarkably, co-stimulation of BCR with TLR9 significantly increased MMP-7 secretion when compared with TLR9 alone (Supplement 5C). BCR co-stimulation with TLR2 showed an increase but was not statistically significant and TLR3 and TLR5 did not show an increase (Supplement 5C).

### MMP-7 promotes the shedding of Syndecan-4

To further understand the function of MMP-7 and since MMPs are known for their ability to cleave syndecan proteins from the cell surface^13–15^, we sought to investigate if MMP-7 mediated shedding of syndecans is a mechanism by which B-lymphocytes may contribute to the acute inflammatory response.

**Figure 8:**
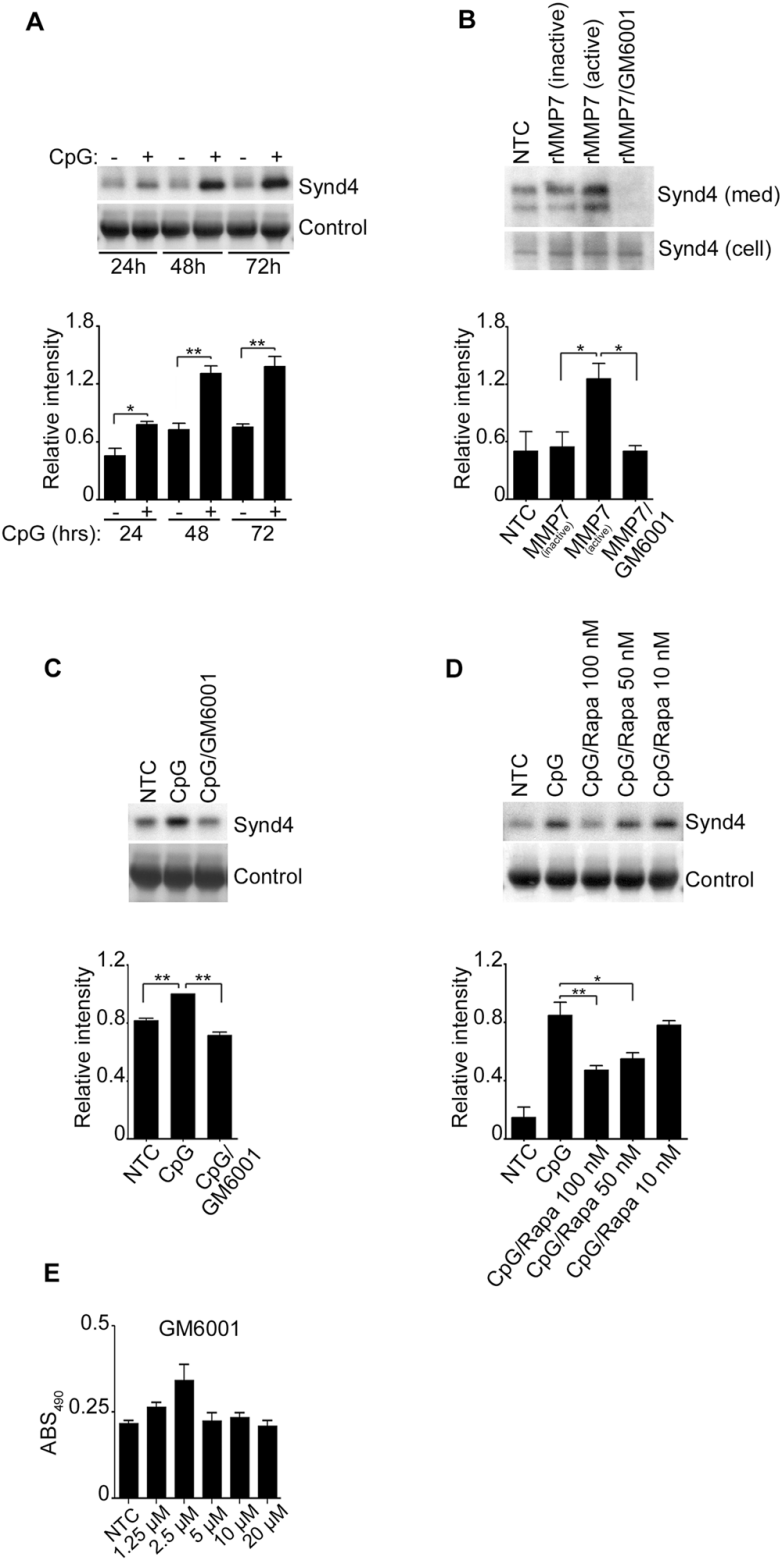
MMP7 mediates the shedding of Syndecan-4 in activated B-lymphocytes. Syndecan-4 was detected by immunoblotting in the cell supernatant after CpG-stimulation for the indicated periods of time (**A**); after incubation with (5 μg/ml) recombinant active and inactive MMP-7 in the presence or absence of (5 μM) MMP-7 inhibitor (GM6001) for 6 hours (**B**); after CpG-stimulation in the presence or absence of GM6001 (**C**)**;** and after CpG-stimulation in the presence of different concentrations of Rapamycin (**D**). Densitometric levels were obtained using ImageJ software and relative protein intensities were normalized against that of loading control. (**E**) Cells were incubated with GM6001 at different concentrations as indicated. XTT was measured after 24 hours. Data are representative of at least three replicate experiments. *p < 0.01, **p < 0.0001.

As mature B-lymphocytes express Syndecan-4^16^, we first investigated if shedding of Syndecan-4 from the cell surface of human peripheral B-lymphocytes was triggered by CpG. As shown in Fig. 8A, CpG stimulation of B-lymphocytes resulted in a time-dependent increase of Syndecan-4 in the cell supernatant indicative of its release into the media. Since primary B-lymphocytes are not suitable for lentivirus infection or transfection with other forms of interfering RNA and to further demonstrate that Syndecan-4 shedding was mediated by MMP-7, cells were treated with recombinant active MMP-7 in the presence or absence of an MMP-7 inhibitor (GM6001). As shown in Fig. 8B, Syndecan-4 was significantly increased after recombinant active MMP-7 was added, but not when the inactive control was used or after the addition of the MMP-7 inhibitor (Fig. 8B). Despite the fact that GM6001 has been previously used as MMP-7 inhibitor, it is possible that it may have an effect on other metalloproteases such as ADAMs and ADAMTSs^17, 18^. While this may be a possibility, our RNA data did not show induction of any of these molecules and therefore they are less likely to participate in the shedding of Syndecan-4 in our system (Supplement 6). To further demonstrate that CpG mediated MMP-7 was responsible for Syndecan-4 shedding, B-lymphocytes were stimulated with CpG in the presence of the MMP-7 inhibitor or Rapamycin. As hypothesized, CPG-stimulated cells demonstrated less Syndecan-4 shedding in the presence of the inhibitors suggesting that MMP-7 mediates the shedding of Syndecan-4 (Fig. 8C and D).

To further understand if MMP-7 participated in B-lymphocyte differentiation, subpopulations of B-lymphocytes were analyzed after CpG and Curdlan for a period of 5 days using well established surface expression markers. Interestingly, no statistical differences were found in the proportion of naïve, unswitched memory, switched memory or plasmablasts before or after stimulation (Supplement 7A,B). CpG stimulation did induce a small fraction of plasmablasts, however we think this is an effect of CpG and not MMP-7 since curdlan-stimulated cells did not induce this population of plasmablasts but still secreted similar levels of MMP-7 (Supplement 7C).

## Discussion

Our study demonstrates that human circulating B-lymphocytes release MMP-7 after stimulation with un-methylated CpG motifs and β-glucans through activation of TLR9 and Dectin-1 receptors, respectively. Interestingly, while both infectious antigens resulted in MMP-7 activation its regulation was receptor-ligand dependent. Whereas both stimuli activated JNK and the transcription factors NF-κB and AP-1, CpG activated the mTOR pathway while β-glucan-activation was mTOR-independent and activated ERK1/2. While the pathways investigated are known to be classically involved in TLR9 and Dectin-1 signaling herein we have shown their novel participation in MMP-7 regulation by circulating human B-lymphocytes. These findings shed further light into the less characterized function of B-lymphocytes as innate immune mediators.

The role of mTOR in response to CpG has been well studied in human B-lymphocytes^19–21^ and while little is known about the role of mTOR in MMP-7 regulation, some connection can be implied from studies done in patients with lymphangioleiomyomatosis (LAM) in which MMP-7 has been found upregulated^22^. LAM is a cystic lung disorder that involves the loss of function of the tuberous sclerosis complex 1 or 2 genes (TSC1 and TSC2) which are well-known suppressors of the mTOR pathway^23–25^. Treatment with mTOR inhibitors is the standard care for patients with symptomatic and progressive disease. Further evidence of the role of mTOR in CpG-mediated MMP-7 can also be inferred from studies in dendritic cells where mTOR activation by TLR9 agonist results in type I interferon (IFN-α/β) secretion^12^. While no direct connection between mTOR and MMP-7 was investigated in those studies, herein we have shown that inhibition of mTOR significantly decrease MMP-7 levels in CpG-stimulated cells, thus providing additional evidence for a link between the mTOR pathway and the regulation of MMP-7 in TLR9 activated B-lymphocytes.

To further understand the regulation of MMP-7, B-lymphocytes were challenged with fungal β-glucans. These experiments showed that B-lymphocytes stimulated *via* Dectin-1, also contributed to the release of MMP-7. However, to our surprise, MMP-7 regulation differed from CpG in that it was mTOR-independent and used ERK1/2 signaling instead. This is important as our data illustrates that both bacterial and fungal triggers are contributors to the circulating pool of MMP-7 and further suggest that different infectious components elicit MMP-7 secretion by using different PRRs and signaling pathways. While the data presented here is focused on human circulating B-lymphocytes our conclusions are supported by studies in gastric cancer where MMP-7 and MMP-2 expression did not correlate with mTOR activation and Rapamycin failed to decrease MMP-7 secretion despite decreasing mTOR phosphorylation^26^. The authors speculated that the observed mTOR independent MMP-7 expression was attributable to the complex regulation of mucosal inflammation in the tissue microenvironment in gastric cancer. Based on our data, an alternative or additional explanation might be the fact that while MMP-7 is upregulated in many types of cancers only a subset of them may be mTOR-dependent as mTOR activation may only be triggered by infectious triggers such as *H*. *pylori* while non-infectious or non-bacterial antigens may not use the mTOR pathway. Additionally, we showed that CpG and β-glucan had a synergistic effect on MMP-7 production. While we acknowledge that this is an *in vitro* system, frequently patients present with co-infections (bacterial and fungal) and it is therefore very plausible that antimicrobial synergy may occur in the clinical setting leading to higher levels of MMP-7 and may explain a lack of response to mTOR inhibition. This is particularly important as other receptors such as TLR2 and BCR co-stimulation with TLR9 also seem to increase MMP-7 secretion. Understanding these mechanisms is therefore essential to successfully modulate MMP-7 expression in the different models of disease.

**Figure 9:**
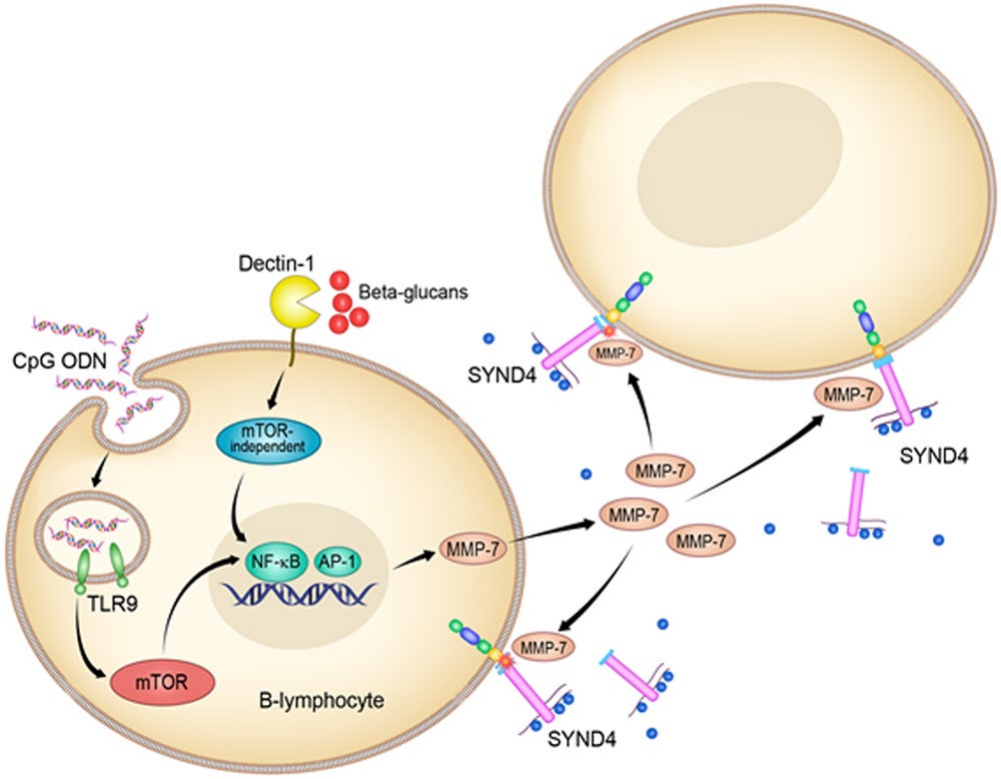
Proposed mechanism of MMP-7-mediated shedding of Syndecan-4 in activated B-lymphocytes. Circulating B-lymphocytes release MMP-7 after CpG and β-glucan stimulation through activation of TLR9 and Dectin-1 receptors, respectively. While CpG-stimulation is Rapamycin sensitive (mTOR-mediated), β-glucan is not (mTOR-independent) indicating different activation pathways. MMP-7 release participates in the shedding of Syndecan-4.

In healthy adults, the activity of MMPs is mostly seen in conditions of tissue remodeling or wound healing as they participate in the breakdown of extracellular matrix proteins such as proteoglycans, fibronectin and elastin. However, MMPs are also known as sheddases as they participate in the cleavage/shedding of the extracellular domain of syndecans converting them into soluble ectodomains that will bind through the heparin sulfate chains to a variety of cytokines, chemokines, collagens and extracellular matrix glycoproteins^2, 27^. By modulating the shedding of syndecan proteins, MMPs indirectly participate in the trafficking of different cytokines and chemokines. Herein, we have shown that recombinant MMP-7, as well as CpG-induced MMP-7, increased the shedding of Syndecan-4 in by B-lymphocytes. Furthermore, we demonstrated that the release of Syndecan-4 into the media was blocked by an MMP-7 inhibitor, suggesting that CpG mediated shedding of Syndecan-4 requires MMP-7. Additionally, we showed that Rapamycin which inhibits MMP-7 also decreased the shedding of Syndecan-4 likely by interfering with MMP-7. While the nature of working with primary human B-lymphocytes does not allow us the use of interference RNA or similar approaches to knock down proteins and we have to rely on chemical inhibitors, our data supports the conclusion that MMP-7 is likely one of the main sheddases for Syndecan-4 since MMP-7 was the only MMP that was found to be induced in our system. Furthermore, MMP-7-mediated cleavage of syndecan proteins, including Syndecan-4, is well established and documented in the literature as is the MMP-7 regulation of chemokine gradients *via* syndecan proteins^3, 13, 28, 29^. Particularly, MMP-7 participates in important functions such as neutrophil activation by modulating syndecan-1/CXCL1 complexes in airway epithelial cells^3^. As MMP-7 is released by activated B-lymphocytes and participates in the shedding of Syndecan-4 expressed on B-lymphocytes, it is also likely to influence the shedding of other syndecans and heparin-like molecules not just from B-lymphocytes, but other neighboring cells (see Fig. 9). However, further investigations are needed to further confirm this model.

It is clear that in the wounded epithelium MMP-7 expression is necessary for recruiting inflammatory cells and facilitating epithelization^30^. However, increased levels of MMP-7 have been shown in aberrant healing processes such as pulmonary fibrosis, especially in patients with idiopathic pulmonary fibrosis, where the levels of MMP-7 seem to correlate with disease severity and prognosis^31–33^. Despite the clear up-regulation of MMP-7 in these patients, the role in the pathogenesis of this disease is not clearly understood. The fact that stimulated B-lymphocytes are an additional source of MMP-7, together with the finding that MMP-7 is not exclusively regulated by the mTOR pathway, may shed some light in the pathophysiology of this and other inflammatory and malignant process where MMP-7 is clearly upregulated^22, 33, 34^.

In summary, we report a new function of B-lymphocytes as contributors to the release MMP-7 and the shedding of Syndecan-4 in response to infectious triggers. MMP-7 secretion is mTOR-dependently and independently regulated depending on the stimulating antigen and PRR triggered. This differential regulation is important and novel and needs to be considered when targeting MMP-7. Together these results further suggest that B-lymphocytes participate in the acute inflammatory process by releasing MMP-7 and controlling the shedding of Syndecan-4, both of which are important regulators of chemokine influx essential for cell activation and recruitment in processes such as tissue repair and inflammation.

## Electronic supplementary material

Supplemental figures
