# Supplementary material for: Microbial Antigens Stimulate Metalloprotease-7 Secretion in Human B-Lymphocytes Using mTOR-Dependent and Independent Pathways
Source: Sci Rep. 2017 Jun 20;7:3869. doi: 10.1038/s41598-017-04199-2 (PMC5478602; doi:10.1038/s41598-017-04199-2)

**Microbial Antigens Stimulate Metalloprotease-7 Secretion in Human B-Lymphocytes Using mTOR-Dependent and Independent Pathways**

Mohamed F. Ali^*^

Harika Dasari*

Virginia P. Van Keulen*

Divi Cornec*

George Vasmatzis^$^

Tobias Peikert^*,#^

Eva M. Carmona^*,#,†^

# *From the Thoracic Diseases Research Unit, the ^#^Division of Pulmonary Critical Care and Internal Medicine, ^$^Department of Medicine and Biomarker Discovery, Center for Individualized Medicine Mayo Clinic and Foundation, Rochester, Minnesota 55905

# †Corresponding Author: Eva M Carmona, MD, PhD, 8-48 Stabile Building, Mayo Clinic, Rochester, Minnesota, 55905, Phone: (507) 284-4162, FAX (507) 284-4521, E-mail: carmona.eva@mayo.edu

**SUPPLEMENTAL FIGURES**

**Supplement 1. Oligonucleotide sequences used in real-time PCR analysis.**

| Gene | Forward | Reverse |
| --- | --- | --- |
| GAPDH | 5’- ACATCGCTCAGACACCATG | 5’- TGTAGTTGAGGTCAATGAAGGG |
| MMP1 | 5’-ATGTGGCTCAGTTTGTCCTC | 5’-GCTTTCTCAATGGCATGGTC |
| MMP2 | 5’-AAGTGGGACAAGAACCAGATC | 5’-GATTCGAGAAAACCGCAGTG |
| MMP3 | 5’-AAGCTCTGAAAGTCTGGGAAG | 5’-CAGGTCCATCAAAAGGGTAAAAG |
| MMP7 | 5’-TTCGATGAGGATGAACGCTG | 5’-GGATCTCCATTTCCATAGGTTGG |
| MMP8 | 5’-TGGGAACGCACTAACTTGAC | 5’-CTGGTGAAGATGAGAGGTGATG |
| MMP9 | 5’-AGACATCGTCATCCAGTTTGG | 5’-GGGACCACAACTCGTCATC |
| MMP10 | 5’-GACTCCACTCACATTCTCCAG | 5’-CCATAAAGCCCAGGTCCAG |
| MMP11 | 5’-AGAGGTTCGTGCTTTCTGG | 5’-TCACATCGCTCCATACCTTTAG |
| MMP12 | 5’-TTTTGGACCTGGATCTGGC | 5’-CCTTTGGATCACTAGAATGGCC |
| MMP13 | 5’-GATGACGATGTACAAGGGATCC | 5’-ACTGGTAATGGCATCAAGGG |
| MMP14 | 5’-TGATGCAGACACCATGAAGG | 5’-TGTTGCCATTTGAGACCCTG |
| TIMP1 | 5’-CCCCTAGCGTGGACATTTATC | 5’-AACAGGATGCCAGAAGCC |
| TIMP2 | 5’-CCCTCTGTGACTTCATCGTG | 5’-GAGATGTAGCACGGGATCATG |
| TIMP3 | 5’-CCTTCTGCAACTCCGACATC | 5’-GCCTCGGTACATCTTCATCTG |
| TIMP4 | 5’-ACGCCTTTTGACTCTTCCC | 5’-TCGATGTAGTTGCACAGATGG |

**Supplement 2. MMPs expression in B-lymphocytes stimulated with CpG.** Quantitative real-time PCR (qPCR) for indicated mRNAs in non-treated cells (B cells) or stimulated with CpG for 24 hours (B cells/CpG). Expression was normalized to GAPDH. **P<0.001*


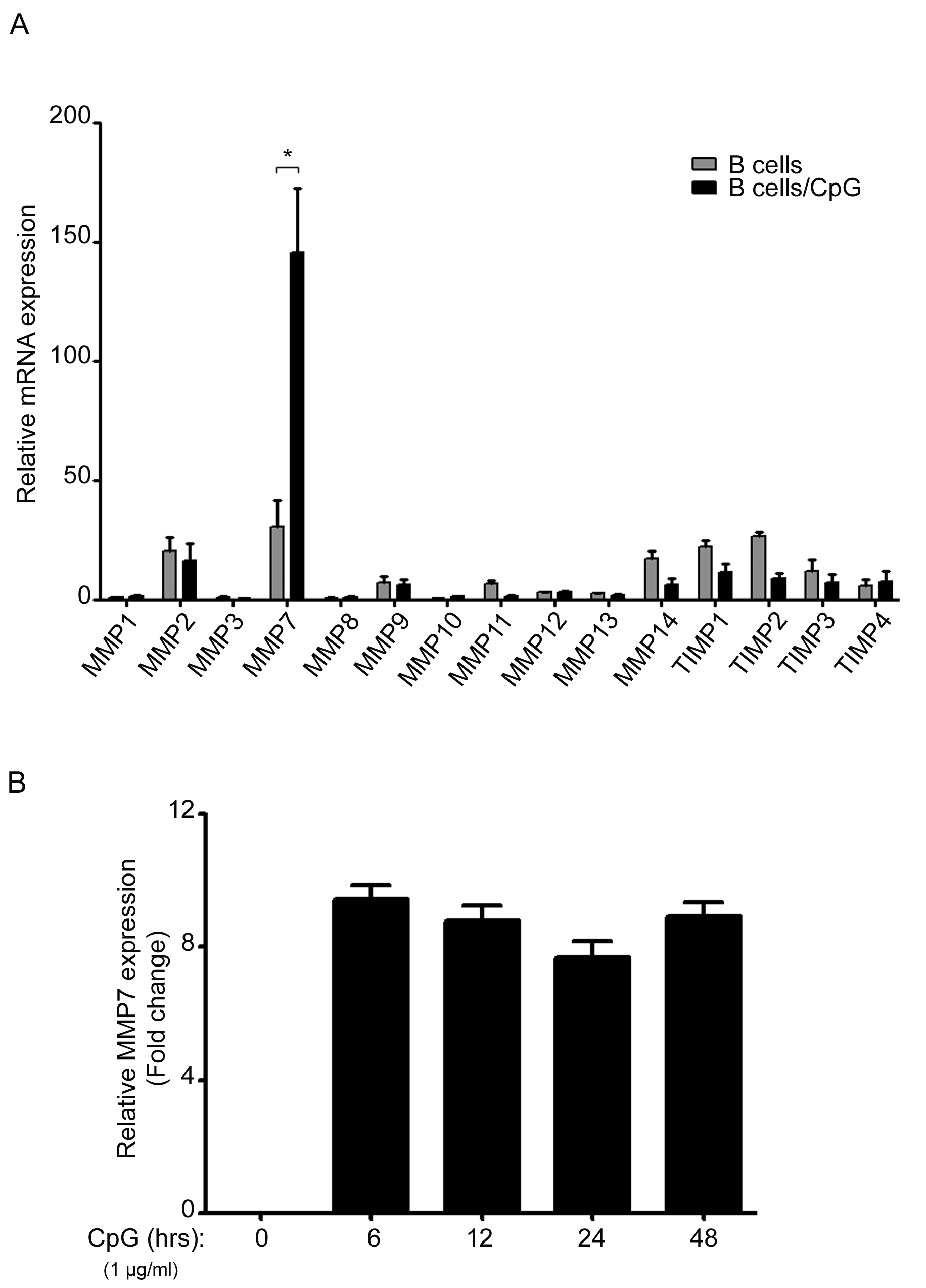


**Supplement 3. MMP7 secretion after CpG stimulation cells sorted by FACS.** MMP-7 was measured by ELISA in the cell supernatant of B-lymphocytes after stimulation with CpG overnight. B-lymphocytes were first isolated by negative selection followed by FACS cell sorting on CD19-positive cells. **p*<0.001 and ***p*<0.0001


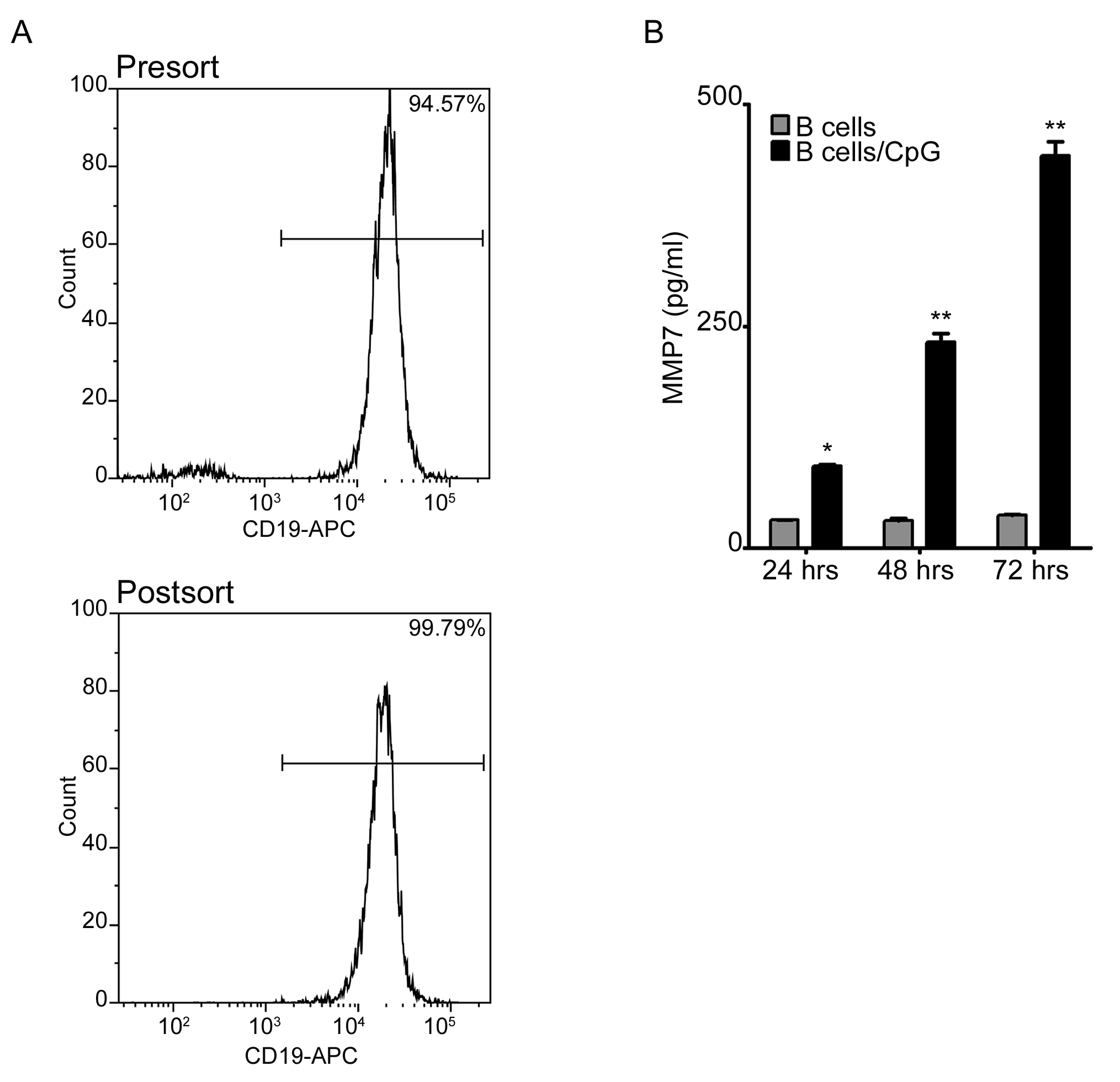


**Supplement 4. TLR and BCR regulation of MMP-7**. B-lymphocytes were isolated by negative selection, stained for 30 minutes with anti-CD20 and anti-CD27 antibodies (BD Pharmingen), and sorted into (CD27^+^ and CD27^-^) as shown [A]. Sorted cells were then plated at 2 x 10^6^/ml and stimulated with CpG and Curdlan. [B] MMP-7 ELISA was measured in the cell supernatant 24 hours later. [C] Neutrophils, B-lymphocytes and PBMCs depleted from B-lymphocytes were obtained from whole heparinized blood. Then cells were plated at a concentration of 4 x 10^6^ cells/ml and stimulated with CpG and Curdlan as indicated. MMP-7 ELISA was then measured in the supernatant 24h later. Data are representative of at least three independent experiments. Ns, not significant.


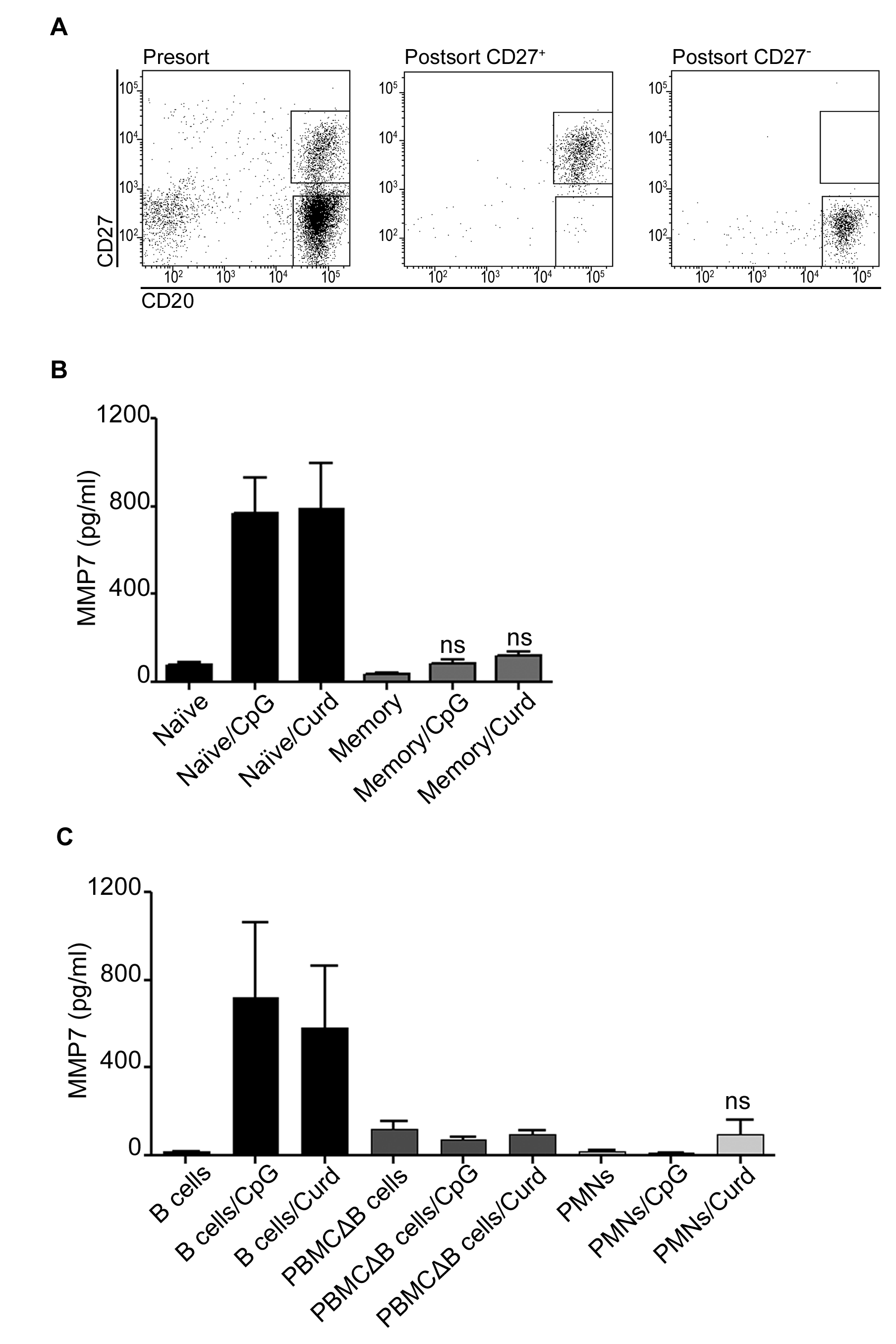


**Supplement 5. MMPs and TIMPS expression in BCR and TLR stimulated B-lymphocytes.** MMP-7 was measured by ELISA in the cells supernatant of B-lymphocytes after different TLR ligand stimulation (TLRL) **[A],** BCR ligand stimulation (BCRL) [B] and TLRL/BCRL [C]. [D] Quantitative real-time PCR (qPCR) for indicated mRNAs in non-treated cells (B cells) or stimulated with BCR ligand for 24 hours (B cells/BCRL). Expression was normalized to GAPDH. Data are representative of at least two independent experiments. **p* <0.006, **p <0.001 and ns, not significant (*p*>0.05).


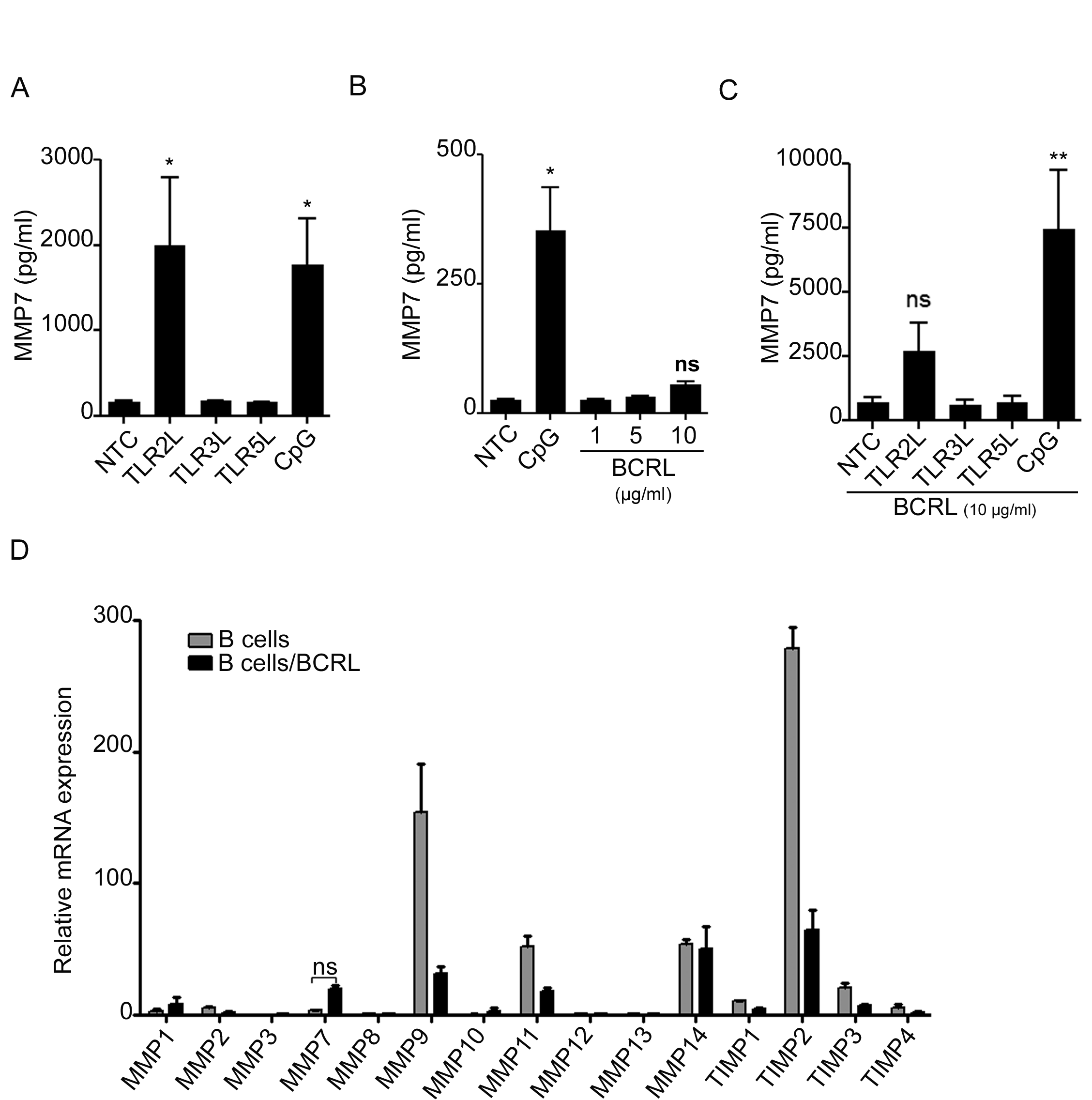


**Supplement 6.** **Heat map of ADAMs and ADAMTSs expression.** B-lymphocytes from 3 different donors were left untreated or stimulated with CpG as indicated. RNA was then isolated. Gene expression profiles were generated using Affimetrix PrimeView chip and analyzed using transcriptome console 3.0. Genes were considered increased from baseline if a change of at least 2-fold or greater was observed.


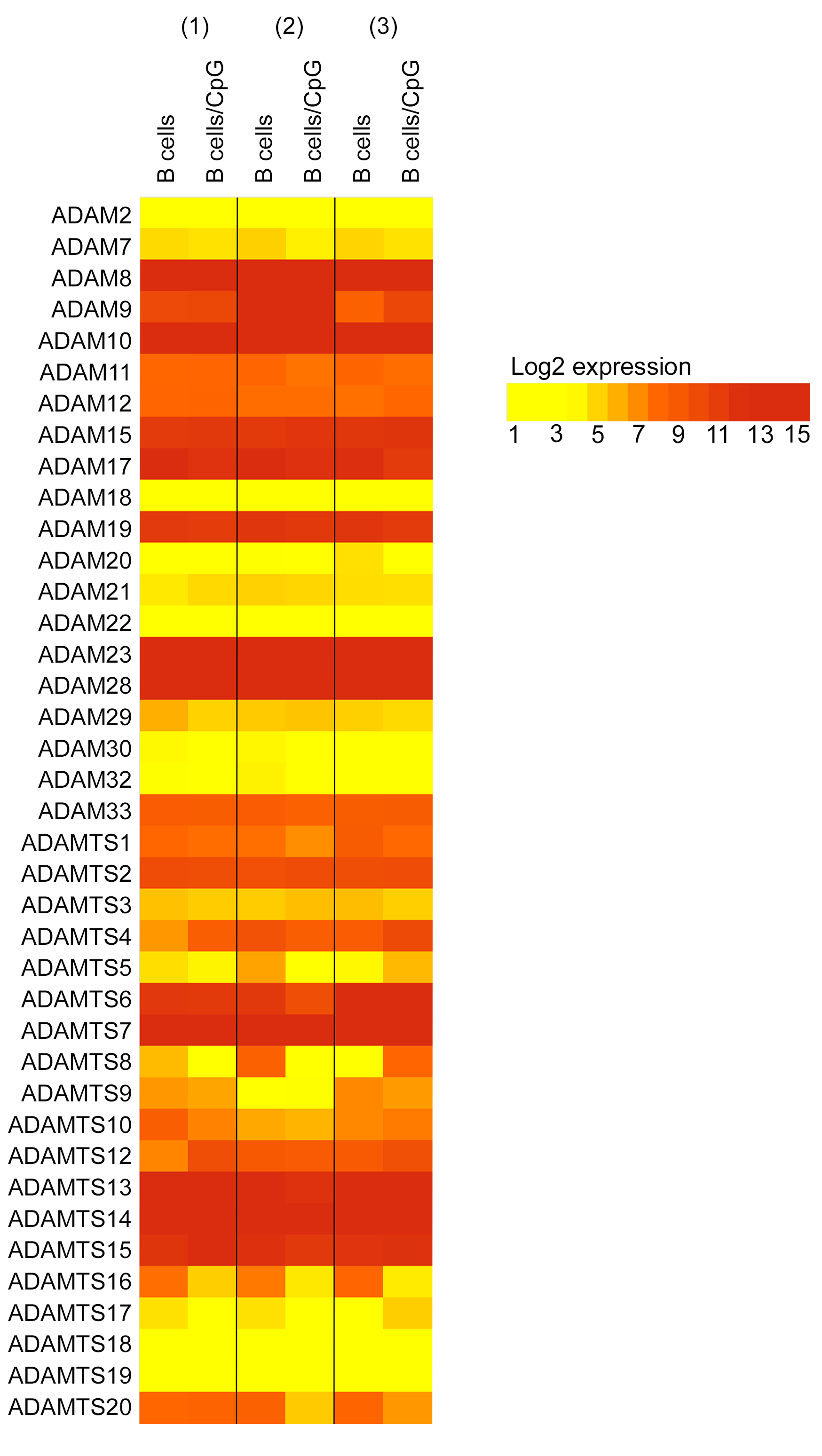


**Supplement 7. Increase in MMP-7 does not associate with changes in B-lymphocyte subtypes.** Peripheral B-lymphocytes (5 x 10^5^ cells per sample) from 3 different donors were treated with CpG and curdlan for 5 days, as indicated. Cells were then washed and incubated with a cocktail of different antibodies (anti-CD19, Anti-Human IgD, anti-CD27, anti-CD38) and analyzed by FACS. [A] Representative gating strategy from one donor. [B] B-lymphocyte subset proportions from the pool of the 3 donors in unstimulated and stimulated cells as indicated. [C] Cell supernatant was collected from the same samples and MMP-7 was measured by ELISA. Data represent a pool of 3 different donors. **p* <0.0002 and ns, not significant (*p*>0.05).


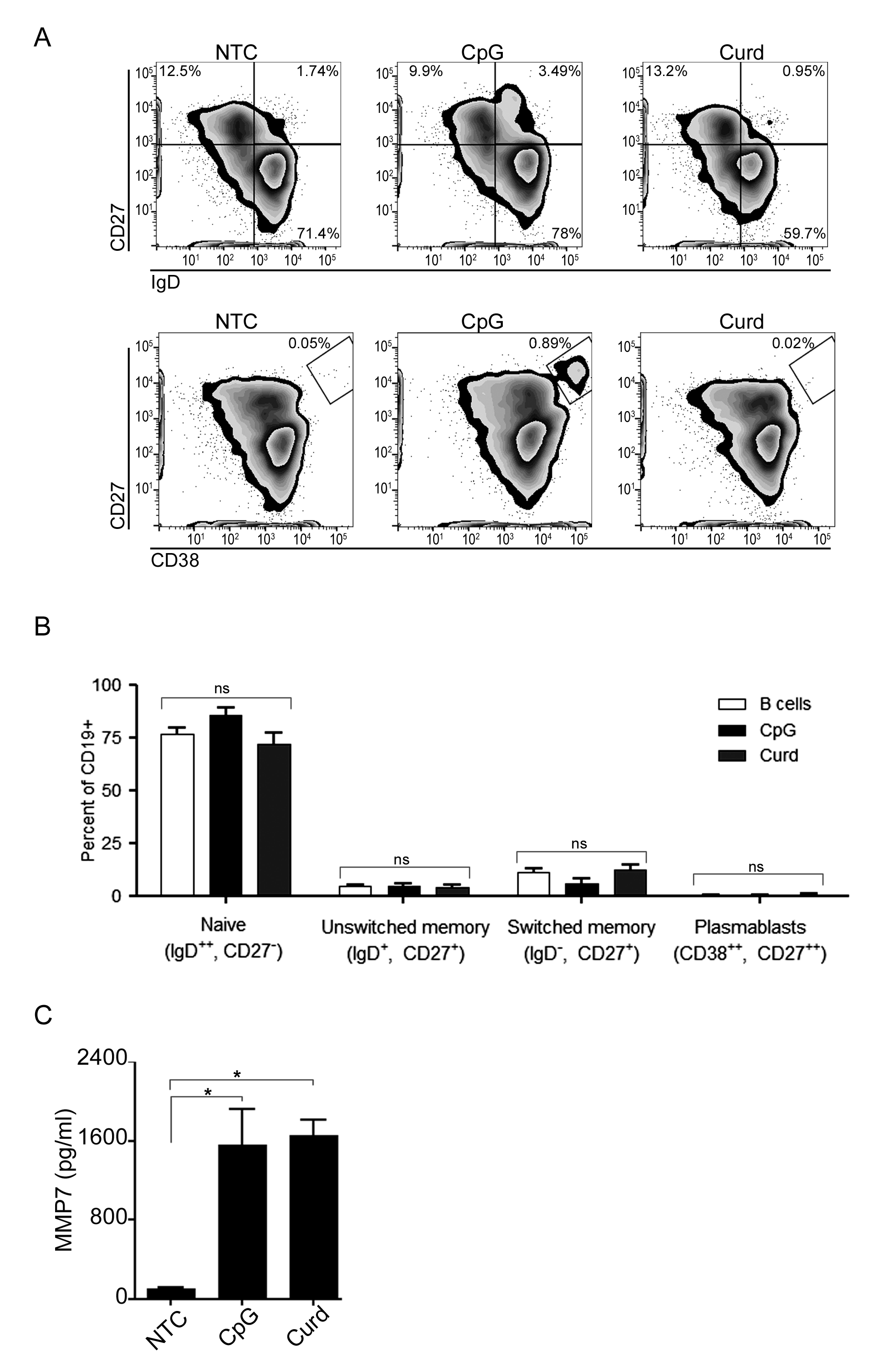

Supplement: Supplementary file 1 — Supplemental figures [file 41598_2017_4199_MOESM1_ESM.doc]
